# A resource description framework (RDF) model of named entity co-occurrences in biomedical literature and its integration with PubChemRDF

**DOI:** 10.1186/s13321-025-01017-0

**Published:** 2025-05-21

**Authors:** Qingliang Li, Sunghwan Kim, Leonid Zaslavsky, Tiejun Cheng, Bo Yu, Evan E. Bolton

**Affiliations:** National Center for Biotechnology Information, National Library of Medicine, National Institutes of Health, Bethesda, MD 20894 USA

**Keywords:** PubChem, RDF, Semantic web, Linked data, Triplestore, SPARQL, Data modeling, PubChemRDF

## Abstract

Named entities, such as chemicals/drugs, genes/proteins, and diseases, and their associations are not only important components of biomedical literature, but also the foundation of creating biomedical knowledgebases and knowledge graphs. This work addresses the challenges of expressing co-occurrence associations between named entities extracted from a biomedical literature corpus in a machine-readable format. We developed a Resource Description Framework (RDF) data model and integrated it into the PubChemRDF resource, which is freely accessible and publicly available. The developed co-occurrence data model was populated into a triplestore with named entities and their associations derived from text mining of millions of biomedical references found in PubMed. The utility of the data model was demonstrated through multiple use cases. Together with meta-data modeling of the references including the information about the author, journal, grant, and funding agency, this data model allows researchers to address pertinent biomedical questions through SPARQL queries and helps to exploit biomedical knowledge in various user perspectives and use cases.

**Supplementary Information:**

The online version contains supplementary material available at 10.1186/s13321-025-01017-0.

## Scientific contribution

The RDF data model developed in this work encodes co-occurrence associations among chemicals, genes, and diseases, derived from biomedical literature. The developed model enables researchers to use SPARQL queries to semantically explore biomedical knowledge and make new discoveries. It also seamlessly links to scientific data in other information resources, improving the usability and accessibility of biomedical data in the Semantic Web.

**Supplementary Information:**

The online version contains supplementary material available at 10.1186/s13321-025-01017-0.

## Introduction

PubChem [1–3] is an open chemical database at the National Center for Biotechnology Information (NCBI), the National Library of Medicine (NLM), the US National Institutes of Health (NIH). Among many tools and services provided by PubChem are the literature knowledge panels [4], which assist users in quickly finding important relationships between chemicals, genes, proteins, and diseases. The literature knowledge panels for a given entity (e.g., a chemical, gene, or protein) display a selected set of “co-occurrence neighbors”, which are defined as any chemicals, genes, proteins, and diseases mentioned together with that entity in the biomedical literature. In addition, the literature knowledge panel provides a sample of PubMed records that co-mention the entity and its selected co-occurrence neighbors. The list of the co-occurrence neighbors and relevant PubMed records can be downloaded for further analysis through the download button in the panel. Note that the use of the term “co-occurrence neighbors” avoids confusion with the existing 2-dimensional (2-D) and 3-dimensional (3-D) chemical structure-based neighbor relationships [5–7] in PubChem. Currently, the underlying data for the knowledge panel is derived from text mining of millions of biomedical references in PubMed, using the LeadMine software [8]. Chemical, gene, protein, and disease entities are extracted from the titles and abstracts of the PubMed records and matched to records in PubChem. For each entity, the most relevant co-occurrence neighbors are identified using statistical analysis and relevance-based sampling. A detailed explanation of the method used to develop the knowledge panel is given in our previous paper [4].

The present paper describes a data model that expresses the named entities and their co-occurrence associations in the Resource Description Framework (RDF) format [9], along with the meta-data modeling of the reference information (including the author, journal, grant, and funding agency). The data model augments the existing RDF-formatted PubChem data (also known as PubChemRDF) [10] and helps find answers to biomedical questions (as demonstrated in a recent study [11]) through SPARQL Protocol and RDF Query Language queries [12]. The present article was originally presented at the 14th International Conference on Semantic Web Applications and Tools for Health Care and Life Sciences (SWAT4HCLS 2023) (https://www.swat4ls.org/workshops/basel2023/) [13]. It was modified from its original version to reflect discussions with scientists during the conference as well as technical updates since made.

## Construction and content

### Co-occurrence data set

The co-occurrence data used in this study was derived from text mining of 36 million PubMed records available on June 6, 2023. The LeadMine software [8] from NextMove Software was used to extract chemicals, genes, proteins, and diseases from the titles and abstracts of the PubMed records. The extracted entities were matched to records in PubChem. Statistical analysis and relevance-based sampling were employed to determine the most relevant co-occurring neighbors for each entity. A comprehensive description of the methodology used to generate the co-occurrence data can be found in our previous work [4].

### RDF data model

The purpose of the model design is (1) to semantically describe the co-occurrence relations of the named entities (i.e., chemicals, genes/proteins, and diseases), which are derived from the biomedical literature, and (2) to take advantage of the existing PubChemRDF graph to address sophisticated biomedical questions in a semantic way through RDF. In this model, the term “compound” is used to indicate a chemical named entity to keep consistency in the naming convention within PubChemRDF. Because the names of proteins and their encoding genes are often used interchangeably in literature, the data model designed in this study does not distinguish between genes and proteins and, instead, treats all of them as genes, unless explicitly specified.

**Fig. 1:**
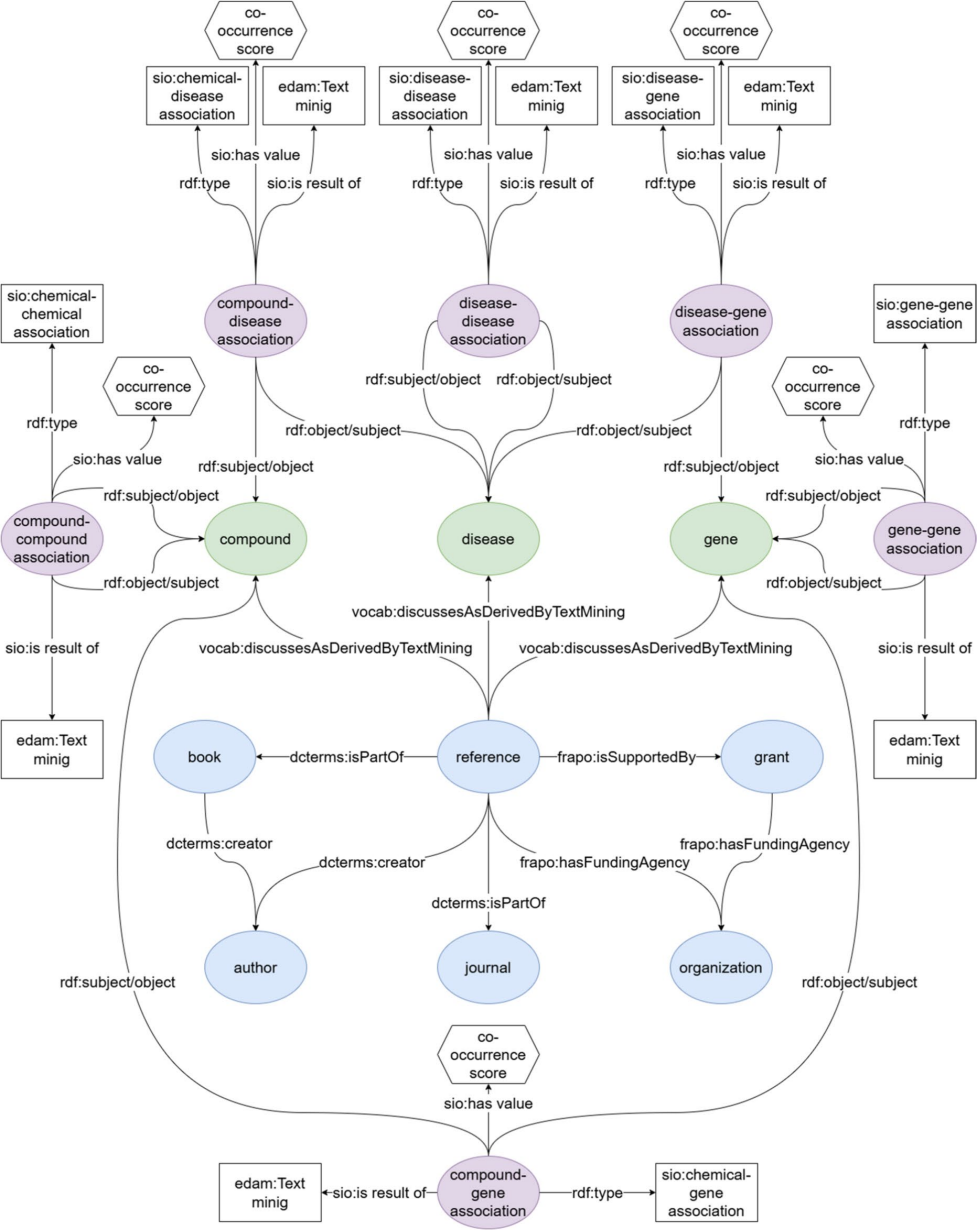
Diagram of RDF co-occurrence modeling of compounds, genes, and diseases, derived from biomedical literature. Blue ovals correspond to biomedical literature (references) and its metadata. Green ovals represent named entities (i.e., compounds, genes, and diseases). Purple ovals indicate the co-occurrence associations between named entities (e.g., compound-disease association, disease-gene association). White rectangles specify the types of individual associations in terms of external ontologies. White hexagons indicate the co-occurrence scores

Figure 1 shows the design of the co-occurrence RDF model. There are five types of nodes in this model:

- Green ovals corresponding to named entities (i.e., compounds, genes, and diseases)
- Blue ovals representing references and their metadata (e.g., author, journal, grant, funding agency)
- Purple ovals for the co-occurrence of named entities (e.g., compound-compound, compound-gene, and compound-disease associations)
- White rectangles describing the characteristic of an association (e.g., text mining and type of associations) used for linking to external ontologies.
- White hexagons representing the co-occurrence score of an association (see below).

The relationships between nodes are defined using the PubChem-specific vocabulary as well as several external ontologies: the Semanticscience Integrated Ontology (SIO) [14]; Funding, Research Administration and Projects Ontology (FRAPO) [15]; Dublin Core Metadata Initiative (DCMI) metadata terms [16]; Publishing Requirements for Industry Standard Metadata (PRISM) vocabularies [17]; and EMBRACE Data And Methods (EDAM) ontology [18].

Named entities used for the RDF data modeling are extracted from PubMed articles through text mining and matched to compounds, genes/proteins, and diseases in PubChemRDF, as described in our previous study [4]. The associations between the PubMed records and the named entities found in them are linked with a predicate, vocab:discussesAsDerivedByTextMining.

When two named entities are identified in a PubMed record, a co-occurrence node is created and linked to the corresponding named entity nodes, with the rdf:subject and rdf:object predicates. The co-occurrence node is also linked to the nodes specifying the nature of the co-occurrence (e.g., the types of co-mentioned named entities and whether they are text-mined or not).

While some named entities are very rare and are co-occurring with just a few other named entities, other entities have thousands of co-occurrence neighbors. For example, the term “neoplasms” co-occurs with over 47,000 compounds, 28,000 genes, and 6000 diseases, which should not be surprising given that cancer has been an intense focus of study for many years. The importance of individual entity relationships is quantified using the co-occurrence score (S_CO_), as explained in our previous study [4]. It can be considered as a variant of the term frequency-inverse document frequency (TF-IDF) score [19–22]. The co-occurrence score values are used to identify a prioritized set of up to 1000 co-occurrence neighbors of each neighbor type (i.e., compounds, genes, and diseases) for a given entity, and the corresponding co-occurrence nodes are created in the model.

It is important to note that the co-occurrence neighboring relationships between entities (e.g., compound-disease) are not symmetrical due to the asymmetry of the co-occurrence scores and the truncation of the neighbor lists. For example, when a disease is one of the top 1000 co-occurrence neighbors of a chemical, there is no guarantee that the chemical is among the top 1000 co-occurrence neighbors of the disease. This asymmetry is reflected in the directed graph in the RDF by means of designating the subject and the object.

Literature data is modeled in reference nodes, which are linked to the nodes representing their metadata (e.g., journal, author, publication date, grant number, funding agency, and MeSH terms [23]), using the relevant terms from FRAPO [15], DCMI [16], and PRISM [17] as predicates. Note that a reference and its funding agency can be connected directly, as well as indirectly via a specific grant. This direct connection between the reference and the funding agency reflects the fact that some scientific articles acknowledge the funding agencies without explicitly mentioning the grant numbers.

The data model developed in this work is described in the Shapes Constraint Language (SHACL) [24] and Shape Expressions (ShEx) formats [25] and made available at the Zenodo repository [26] for archival purposes. The up-to-date PubChemRDF schema in different formats is also available on the PubChemRDF Schema page (https://pubchem.ncbi.nlm.nih.gov/docs/rdf-schema).

### Uniform resource identifiers for named entities

The Uniform Resource Identifiers (URIs) for chemicals and diseases are based on PubChem compound identifiers (CIDs) and disease identifiers (DZIDs), respectively. For example, the following are the URIs for indomethacin (CID3175) and inflammation (DZID8173):

- http://rdf.ncbi.nlm.nih.gov/pubchem/compound/CID3715 (for indomethacine)
- http://rdf.ncbi.nlm.nih.gov/pubchem/disease/DZID8173 (for inflammation)

The URIs for genes are based on their gene symbols. For example, the URI for the Breast Cancer 2 (BRCA2) gene is:

- http://rdf.ncbi.nlm.nih.gov/pubchem/gene/brca2

It should be emphasized that the gene symbols in the URIs are standardized to lowercase. This choice is to mitigate the ambiguity arising from varying capitalization of the symbols of orthologous genes across species (e.g., “BRCA2” for human, “Brca2” for house mouse, and “brca2” for zebrafish) and the frequent lack of organism information for the gene entities extracted from scientific articles. It is also worth mentioning that some gene symbols contain special characters that are not compatible with the URI syntax, such as a space (e.g., “GAMMA CA1”), an “@” sign (e.g., “HOXA@”), and parentheses (e.g., “L(3)MBT”). To address this issue, all special characters in gene symbols, except for “/”, “-”, “_”, “:”, and “.”, were percent-encoded (e.g., “%20” for a space, “%40” for an “@” sign, “%28” and “%29” for opening and closing parentheses), complying with the RFC 3987 standards on the Internationalized Resource Identifiers (IRIs) [27].

### Automated pipeline for regular updates

As explained in our previous paper [4], an automated pipeline was developed to extract named entities and match them with records in PubChem. The resulting data are converted to the co-occurrence RDF through another automated pipeline and released together with other PubChemRDF data [10, 28] on a monthly basis.

## Utility and discussion

The PubChemRDF data, including the co-occurrence RDF data, are freely available for bulk download on the PubChem File Transfer Protocol (FTP) site (https://ftp.ncbi.nlm.nih.gov/pubchem/RDF/). The PubChemRDF data are partitioned by subdomain [29]. A subdomain refers to all RDF triples that have the same type of entity as their subjects. For example, the compound subdomain contains all triples whose subject is a compound record in PubChem. The RDF data for each subdomain is stored in its own subdirectory. This allows users to download only the desired data, rather than downloading all RDF data. The co-occurrence RDF data are organized into the PubChemRDF “cooccurrence” subdomain. The RDF triples encoding the PubMed articles and their associated meta data are organized into the “reference”, “book”, “journal”, “author”, “grant”, and “organization” subdomains.

In this section, we present several use cases of the co-occurrence RDF resource, along with SPARQL query examples. The prefixes used in the examples are summarized in Table 1. Here, these use cases assume that the co-occurrence RDF resource and PubChemRDF data are loaded into the RDF triplestore of Virtuoso [30]. Running the query examples shown in the paper requires at least five PubChemRDF subdomain data (cooccurrence, reference, compound, gene, and disease). It is possible to load these data into other RDF triplestores or RDF-aware graph databases, such as Apache Jena (https://jena.apache.org). In addition, while both the co-occurrence RDF and PubChemRDF data are routinely updated, the data used in these use cases are from named entities extracted from articles indexed in PubMed as of June 6, 2023, and archived in the Zenodo Repository (10.5281/zenodo.10126725) [26], along with the query examples in plain text format, the machine-readable RDF schema in SHACL and ShEx, and the scripts used for RDF data validation. The latest version of the cooccurrence RDF data and other PubChemRDF data are available at the PubChem FTP site (https://ftp.ncbi.nlm.nih.gov/pubchem/RDF/).

**Table 1:** Prefixes used in the SPARQL query examples (Figs. 2 to 9), along with their namespaces and short descriptions

Prefix	Internalized resource identifier (IRI)	Description
bao	http://www.bioassayontology.org/bao#	BioAssay Ontology (BAO)
compound	http://rdf.ncbi.nlm.nih.gov/pubchem/compound/	PubChem Compound subdomain
dcterms	http://purl.org/dc/terms/	Dublin Core Metadata Initiative (DCMI) metadata terms
disease	http://rdf.ncbi.nlm.nih.gov/pubchem/disease/	PubChem Disease subdomain
gene	http://rdf.ncbi.nlm.nih.gov/pubchem/gene/	PubChem Gene subdomain
obo	http://purl.obolibrary.org/obo/	Open Biological and Biomedical Ontology (OBO) Foundry
prism	http://prismstandard.org/namespaces/basic/3.0/	Publishing Requirements for Industry Standard Metadata (PRISM) vocabularies
rdf	http://www.w3.org/1999/02/22-rdf-syntax-ns#	RDF
sio	http://semanticscience.org/resource/	Semanticscience Integrated Ontology (SIO)
skos	http://www.w3.org/2004/02/skos/core#	Simple Knowledge Organization System (SKOS)
taxonomy	http://rdf.ncbi.nlm.nih.gov/pubchem/taxonomy/	PubChem Taxonomy subdomain
up	http://purl.uniprot.org/core/	UniProt RDF
vocab	http://rdf.ncbi.nlm.nih.gov/pubchem/vocabulary#	PubChem vocabulary

### Use case 1: diseases co-occurring with a chemical

**Fig. 2:**
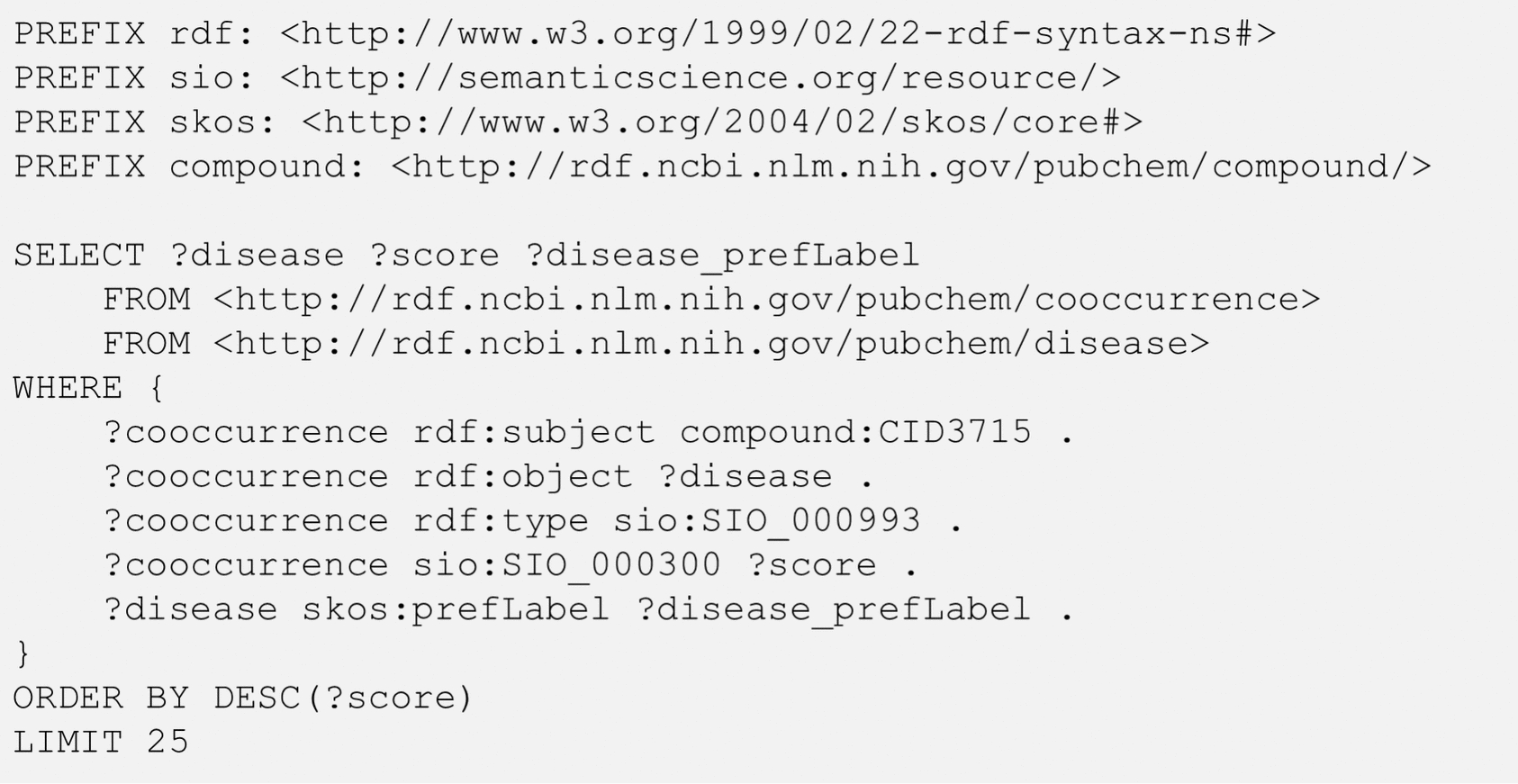
SPARQL query to retrieve the top-25 diseases commonly mentioned with indomethacin (CID 3715) in literature. The SIO terms SIO_000993 and SIO_000300 mean “chemical-disease association” and “has a value”, respectively

The simplest use case of the co-occurrence RDF is to retrieve named entities commonly mentioned with a query entity in PubMed articles (e.g., diseases or genes/proteins co-occurring with a chemical). As an example, Fig. 2 shows the SPARQL query that retrieves the top-25 diseases mentioned together with indomethacin (CID 3715) in terms of their co-occurrence scores [4]. The diseases returned from the query are listed in Table 2, along with their co-occurrence scores and preferred disease names. The preferred disease name was retrieved from the PubChemRDF disease subdomain (with the term prefLabel in the Simple Knowledge Organization System (SKOS) [31] as a predicate).

**Table 2:** Top-25 diseases commonly mentioned with indomethacin (CID 3715), along with their co-occurrence scores (S_CO_), returned from the SPARQL query in Fig. 2

S_CO_^*a*^	Preferred disease name
59,500	Inflammation
56,896	Ulcer
52,223	Edema
42,861	Ductus Arteriosus, Patent
37,799	Stomach Ulcer
34,888	Premature Birth
34,474	Pain
29,202	Hypertension
23,186	Arthritis
22,239	Drug-Related Side Effects and Adverse Reactions
21,653	Arthritis, Rheumatoid
21,161	Infections
21,049	Neoplasms
20,691	Blood Platelet Disorders
20,034	Hypotension
18,340	Depressive Disorder
18,254	Hemorrhage
17,526	Arthritis, Experimental
16,984	Headache
16,940	Rheumatic Diseases
15,547	Hypoxia
14,776	Fever
14,765	Ischemia
12,514	Osteoarthritis
12,073	Bartter Syndrome

^*a*^Derived from the values computed from Formula (3) in Reference [4] by multiplying by 100 and rounding to the nearest integer

The most commonly occurring disease entity with indomethacin in the biomedical literature is “inflammation”, followed by “ulcer”. It reflects the fact that indomethacin is a non-steroidal anti-inflammatory drug (NSAID) and that NSAIDs’ common side effects include stomach ulcer. In essence, this query performs the same data retrieval task used to create the chemical-disease co-occurrence knowledge panel, available on the Summary page of indomethacin (https://pubchem.ncbi.nlm.nih.gov/compound/3715#section=Chemical-Disease-Co-Occurrences-in-Literature). It is noteworthy that, while the co-occurrence RDF allows the user to get up to 1000 co-occurrence neighbors (as explained in the *Construction and Content* section), the knowledge panel on the Compound Summary page only provides a maximum of 25 co-occurrence neighbors.

As explained in our previous study [4], the disease entities in the co-occurrence data were matched to MeSH headers and supplementary concepts. The preferred names of the diseases in Table 2 were the preferred terms of the MeSH records matched to those diseases. For example, the disease “Ductus Arteriosus, Patent” corresponds to MeSH Unique ID D004374 (https://meshb-prev.nlm.nih.gov/record/ui?ui=D004374). The definition of the disease can also be found on the corresponding MeSH record page.

### Use case 2: references that co-mention a particular chemical–disease pair

**Fig. 3:**
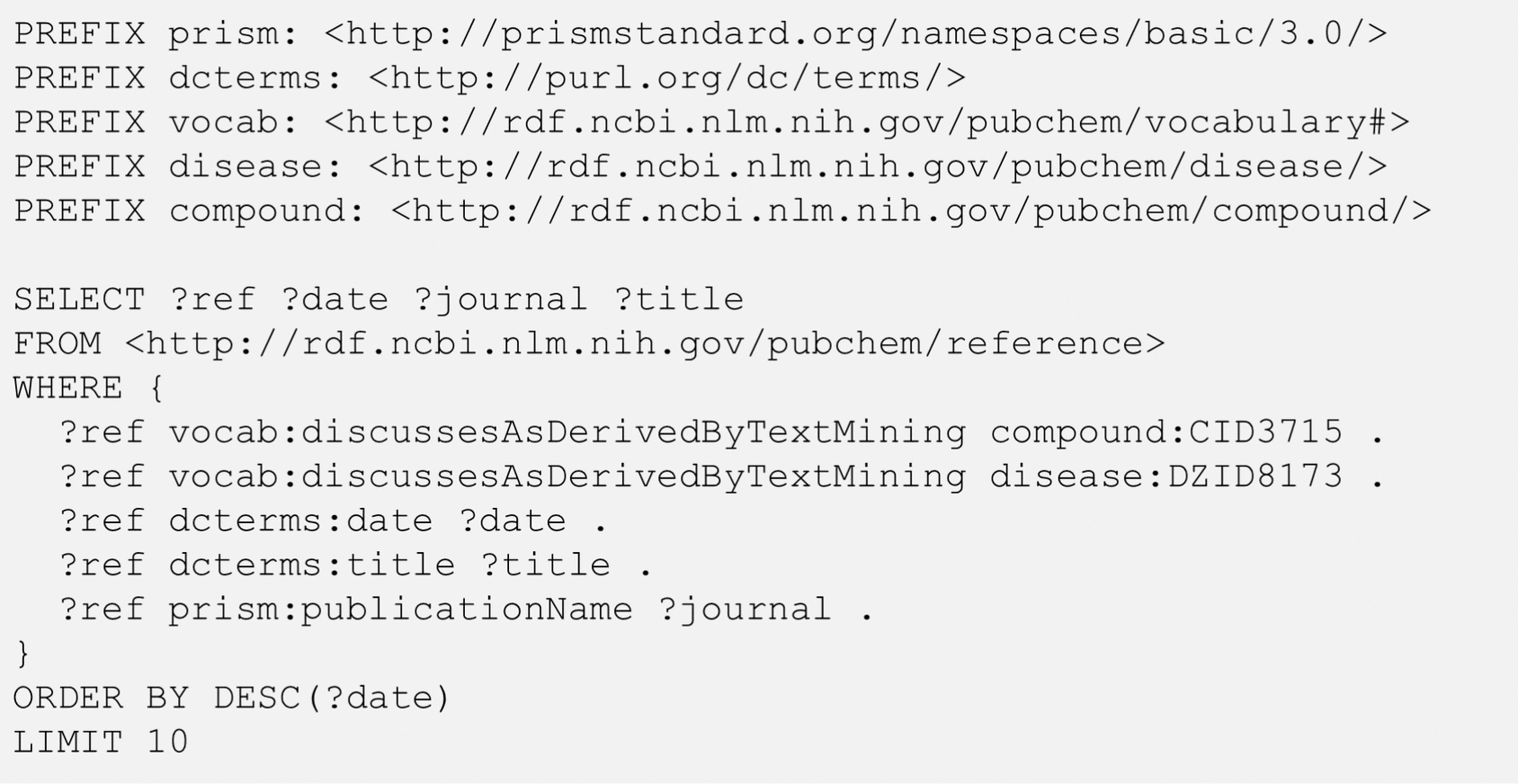
SPARQL query to get the ten most recent references that mention indomethacin (CID3715) and inflammation (DZID8173) together

It is noteworthy that the diseases in Table 2 are related to the input chemical (indomethacin) in different contexts. For example, indomethacin is used to treat inflammatory diseases like arthritis, while it is also known to cause stomach ulcer (as a side effect). To understand the context of the relationship between two entities, it is often necessary to get relevant articles that mention them together. The SPARQL query for this task is shown in Fig. 3, with the indomethacin–inflammation pair as an example.

In the query for Use Case 2, while the named entities are specified using the PubChem-specific vocabulary (vocab:discussesAsDerivedByTextMining), the external vocabularies from DCMI [16] and PRISM [17] are used to get the metadata for the reference (i.e., date, title, and journal). The result of the query is shown in Table 3.

**Table 3:** Ten recent PubMed records that mention indomethacin and inflammation together, returned from the SPARQL query in Fig. 3

Date	Journal	Title
12/31/2022	Pharm. Biol.	Gastroprotective effects of water extract of domesticated *Amauroderma rugosum* against several gastric ulcer models in rats
7/20/2022	Macromol. Biosci.	A Straightforward Approach Towards Antibacterial and Anti-Inflammatory Multifunctional Nanofiber Membranes with Sustained Drug Release Profiles
7/1/2022	Environ. Toxicol.	Protective effect of lupeol on arthritis induced by type II collagen via the suppression of P13K/AKT signaling pathway in *Sprague dawley* rats
7/1/2022	Biomed. Pharmacother.	AMPK/mTOR-driven autophagy & Nrf2/HO-1 cascade modulation by amentoflavone ameliorates indomethacin-induced gastric ulcer
6/25/2022	Int. J. Pharm.	Chitosan/sulfobutylether-β-cyclodextrin based nanoparticles coated with thiolated hyaluronic acid for indomethacin ophthalmic delivery
6/14/2022	Prostaglandins & Other Lipid Mediators	Post-mortem changes of prostanoid concentrations in tissues of mice: Impact of fast cervical dislocation and dissection delay
6/7/2022	J. Dairy Sci.	Induction of leaky gut by repeated intramuscular injections of indomethacin to preweaning Holstein calves
6/3/2022	Nat. Prod. Res.	*Ranunculus* species suppress nitric oxide production in LPS-stimulated RAW 264.7 macrophages
6/1/2022	Biomed. Pharmacother.	Role of ADMA/DDAH-1 and iNOS/eNOS signaling in the gastroprotective effect of tadalafil against indomethacin-induced gastric injury
6/1/2022	Drug Des. Devel. Ther.	Evaluation of Different Surface Coating Agents for Selenium Nanoparticles: Enhanced Anti-Inflammatory Activity and Drug Loading Capacity

### Use case 3: diseases implicitly related to a chemical via genes

**Fig. 4:**
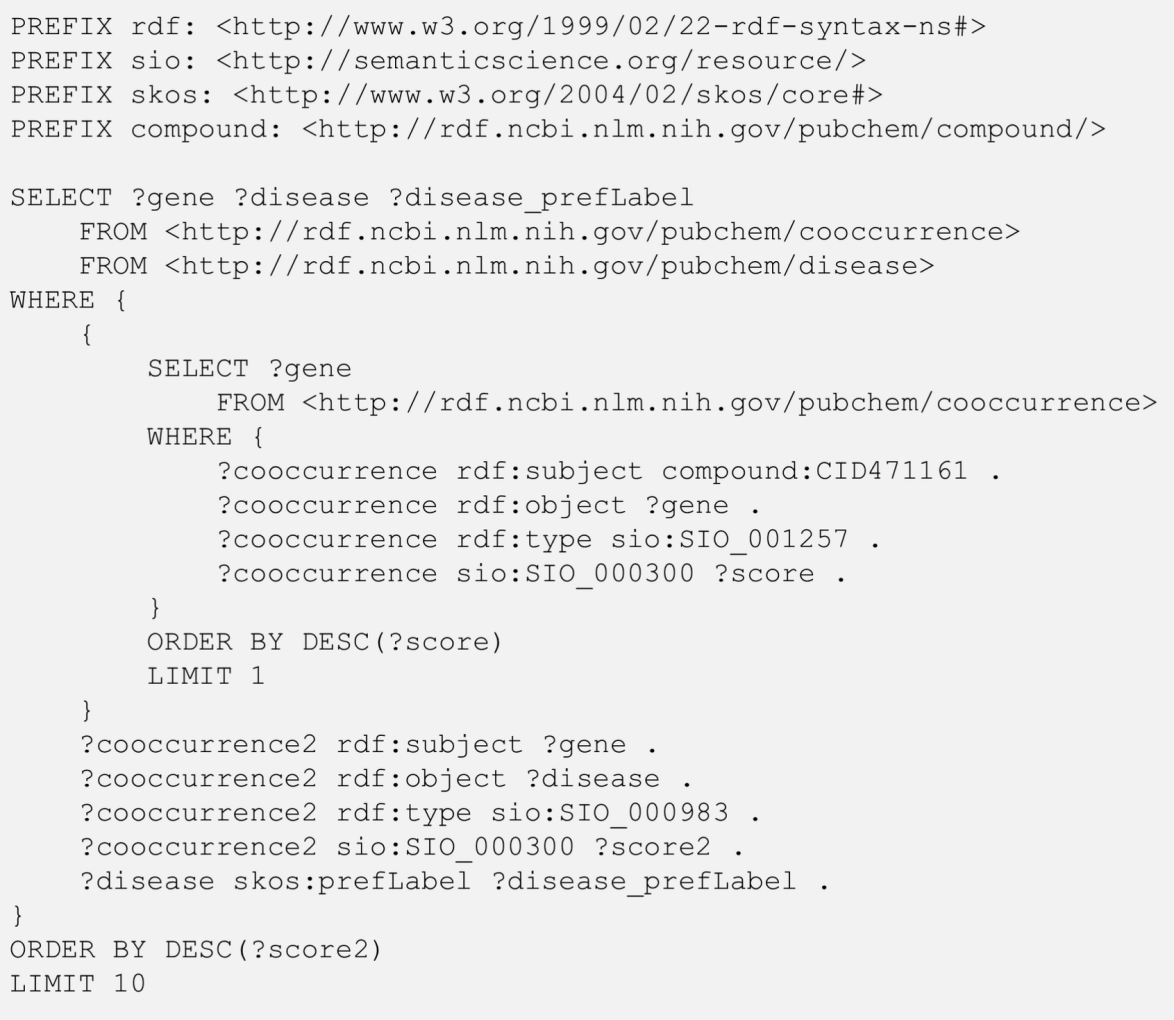
SPARQL query to get the top-10 diseases associated with the gene most commonly mentioned with maribavir (CID471161). The SIO terms SIO_001257, SIO_000983, and SIO_000300 mean “chemical-gene association”, “gene-disease association”, and “has value”, respectively

Use Case 3 intends to find diseases related to a chemical via genes, by first identifying genes commonly mentioned with the query chemical and then retrieving diseases co-occurring with those genes. In this Use Case, while some of the resulting diseases may already be mentioned together with the query chemical in scientific articles, others may not. This implicit relationship can serve as a good starting point to formulate a new hypothesis to test in future studies. Figure 4 shows the SPARQL query with maribavir (CID 471161) as an example.

Maribavir is an antiviral drug approved in 2021 by the US Food and Drug Administration (FDA) for the treatment of posttransplant cytomegalovirus (CMV) infection. Because of its short history, this drug has not been mentioned in many papers, compared to old drugs introduced in the market decades ago (e.g., indomethacin). Table 4 shows the diseases retrieved from the query. The gene most mentioned together with maribavir is the protein kinase, X-linked (PRKX) gene. While some of the diseases co-occurring with PRKX are directly co-mentioned with maribavir in literature, other diseases, including “genetic translocation”, “depressive disorder”, “ischemia”, and “neurodegenerative diseases”, have not appeared with maribavir in PubMed records, implying implicit associations between maribavir and these diseases via the PRKX gene.

**Table 4:** Top-10 diseases associated with the protein kinase, X-linked (PRKX) gene, which is the most co-occurring gene with maribavir (CID 471161)

Preferred disease name	Co-occurring with maribavir
Neoplasms	Yes
Translocation, Genetic	No
Carcinogenesis	No
Polyploidy	Yes
Infections	Yes
Inflammation	Yes
Depressive Disorder	No
Drug-Related Side Effects and Adverse Reactions	Yes
Ischemia	No
Neurodegenerative Diseases	No

The diseases were returned from the SPARQL query in Fig. 4

### Use case 4: chemicals co-mentioned with multiple genes

Drug discovery projects aim to identify drug molecules that can selectively bind to a desired protein target while avoiding other macromolecules that could lead to unwanted off-target side effects. Therefore, the understanding of the interaction of a chemical with multiple genes and proteins is important in the development of safe and effective treatments. Especially, this understanding is even more crucial for the development of multi-target drugs (which can bind to multiple proteins associated with a disease) or selective drugs (which can selectively bind to a desired protein target over other structurally and functionally related proteins).

**Fig. 5:**
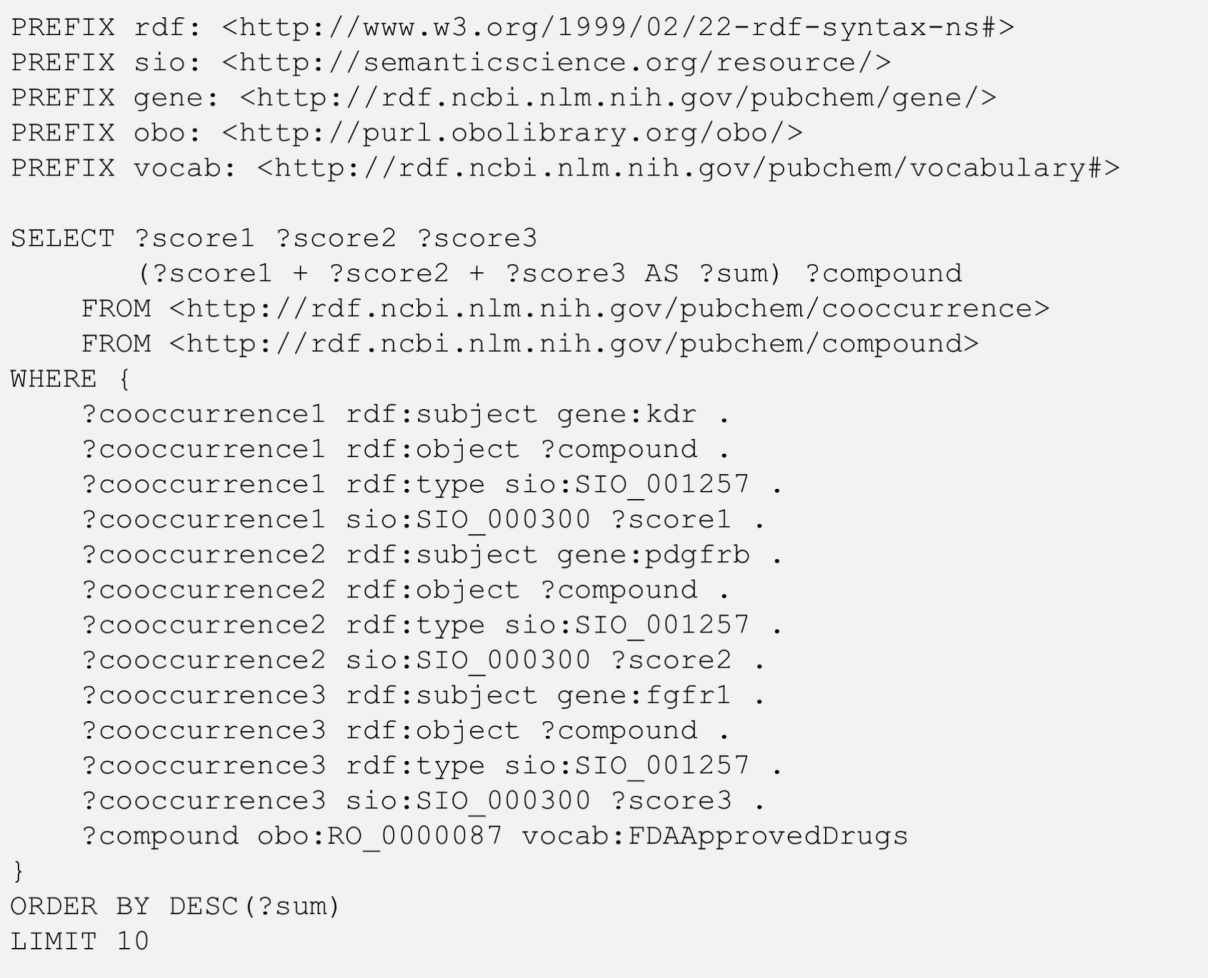
SPARQL query to get the top-10 FDA-approved drugs co-mentioned with three genes: kinase insert domain receptor (KDR), platelet-derived growth factor receptor beta (PDGFRB), and fibroblast growth factor receptor 1 (FGFR1). The SIO terms SIO_001257 and SIO_000300 mean “chemical-gene association” and “has value”, respectively. The RO term, RO_0000087, means “has role”

Figure 5 demonstrates how to retrieve chemicals co-mentioned with three genes: kinase insert domain receptor (KDR) (also known as vascular endothelial growth factor receptor (VEGFR)), platelet-derived growth factor receptor beta (PDGFRB), and fibroblast growth factor receptor 1 (FGFR1). The SIO_001257 predicate (“chemical-gene association”) is used to indicate the relationship between the subjects (the three genes) and the objects (compounds co-mentioned with the genes). The PubChem vocabulary term “FDAApprovedDrugs” is used to retrieve only the compounds that are FDA-approved drugs. The hit list is ordered by the sum of co-occurrence scores for the compound and respective genes and only the top ten compounds with the largest sums are shown using the LIMIT keyword.

Table 5 shows the ten compounds returned from the query in Fig. 5. Seven of them are well-known anti-cancer drugs, as implied in their names. For example, the suffix -*tinib* (in sunitinib, cabozantinib, imatinib, and imatinib mesylate) indicates that these drugs are tyrosine kinase inhibitors. The suffixes -*rafenib* (in sorafenib) and -*anib* (in semaxanib and vandetanib) are used for Raf (rapidly accelerated fibrosarcoma) kinase inhibitors and angiogenesis inhibitors, respectively. It is noteworthy that the hits in Table 5 include tyrosine and oxygen: this is understandable, considering that the hits include tyrosine kinase inhibitors and that angiogenesis inhibitors block the growth of blood vessels that supply oxygen and nutrients to tumor cells.

**Table 5:** Top-10 chemicals co-mentioned with three genes, kinase insert domain receptor (KDR), platelet-derived growth factor receptor beta (PDGFRB), and fibroblast growth factor receptor 1 (FGFR1), returned from the SPARQL query in Fig. 5 along with their co-occurrence scores (S_CO_)

S_CO_			ΣS_CO_	CID	Chemical name
KDR	PDGFRB	FGFR1			
17470	6907	722	25099	5329102	Sunitinib
2713	19759	2267	24739	5291	Imatinib
17213	6202	1266	24681	216239	Sorafenib
8369	5206	3862	17437	6057	L-tyrosine
10794	2119	1611	14524	638015	all-trans-retinal
806	11094	664	12564	123596	Imatinib mesylate
9887	463	289	10639	5329098	Semaxanib
10117	164	164	10445	25102847	Cabozntinib
9717	395	113	10225	3081361	Vandetanib
7620	926	783	9329	977	Oxygen

**Fig. 6:**
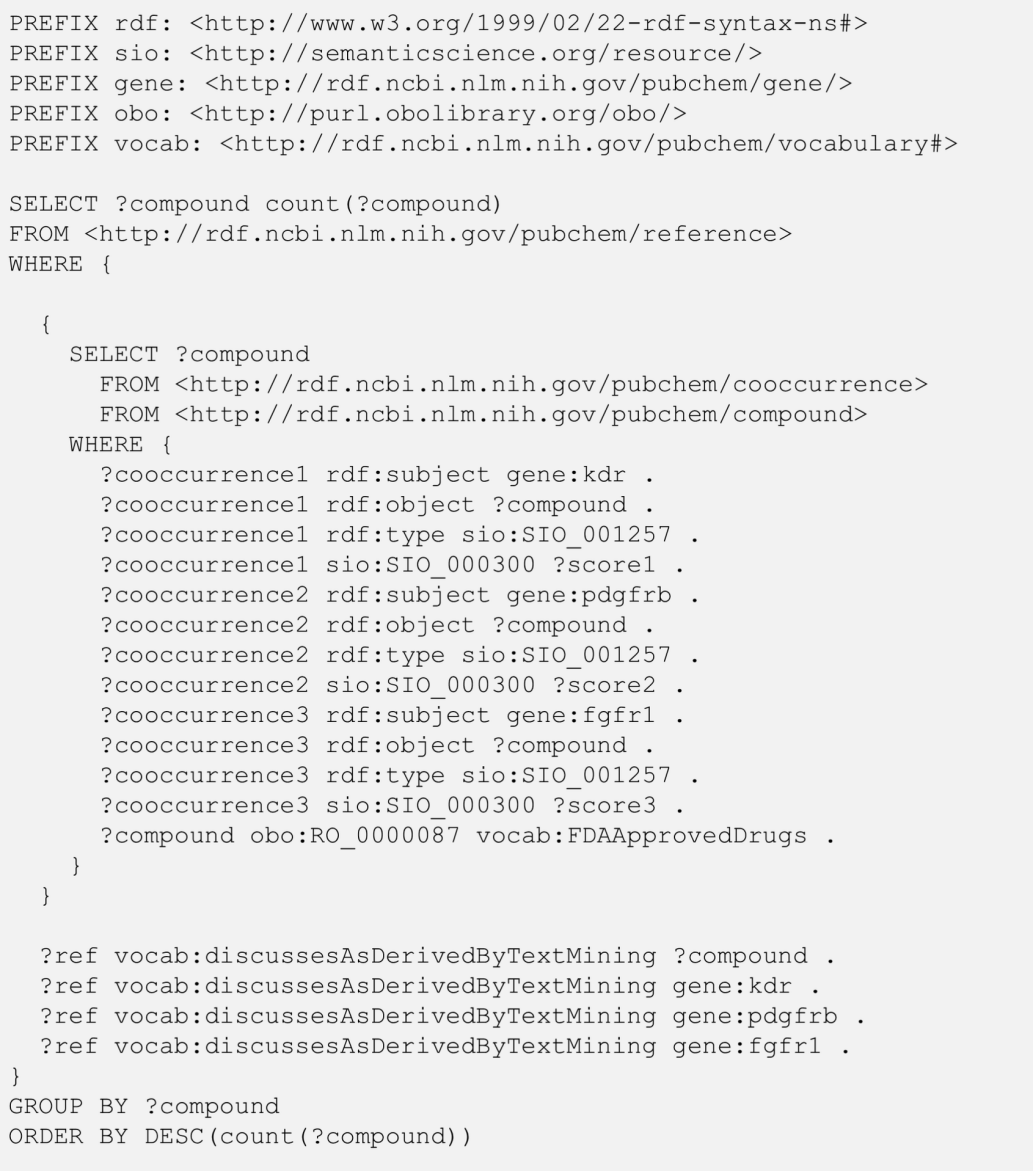
SPARQL query to get the number of references that simultaneously mention an FDA-approved drug and the three genes: kinase insert domain receptor (KDR), platelet-derived growth factor receptor beta (PDGFRB), and fibroblast growth factor receptor 1 (FGFR1). The SIO terms SIO_001257 and SIO_000300 mean “chemical-gene association” and “has value”, respectively. The RO term, RO_0000087, means “has role”

While the query in Fig. 5 finds compounds co-mentioned with the three genes in scientific articles, it does not necessarily mean that the four entities (*i.e.*, a returned chemical and the three genes) have ever been mentioned in a single article. Rather, it is more likely that the chemical is mentioned with one gene in one paper, the second gene in another paper, and the last gene in yet another paper. In contrast, the co-occurrence of the four entities in a single paper is a very rare event, as demonstrated using the query shown in Fig. 6, which retrieves the number of papers that co-mention all four entities together (e.g., a drug and the KDR, PDGFRB, and FGFR1 genes). This example uses a nested query (also known as a subquery). The inner query gets the compounds mentioned with the three genes, which are identical to those returned from Fig. 5. These compounds are used in the outer query, which retrieves articles that co-mentions one of the compounds and all three genes. The vocab:discussesAsDerivedByTextMining predicate is used in the outer query to specify papers and the entities mentioned in them.

**Fig. 7:**
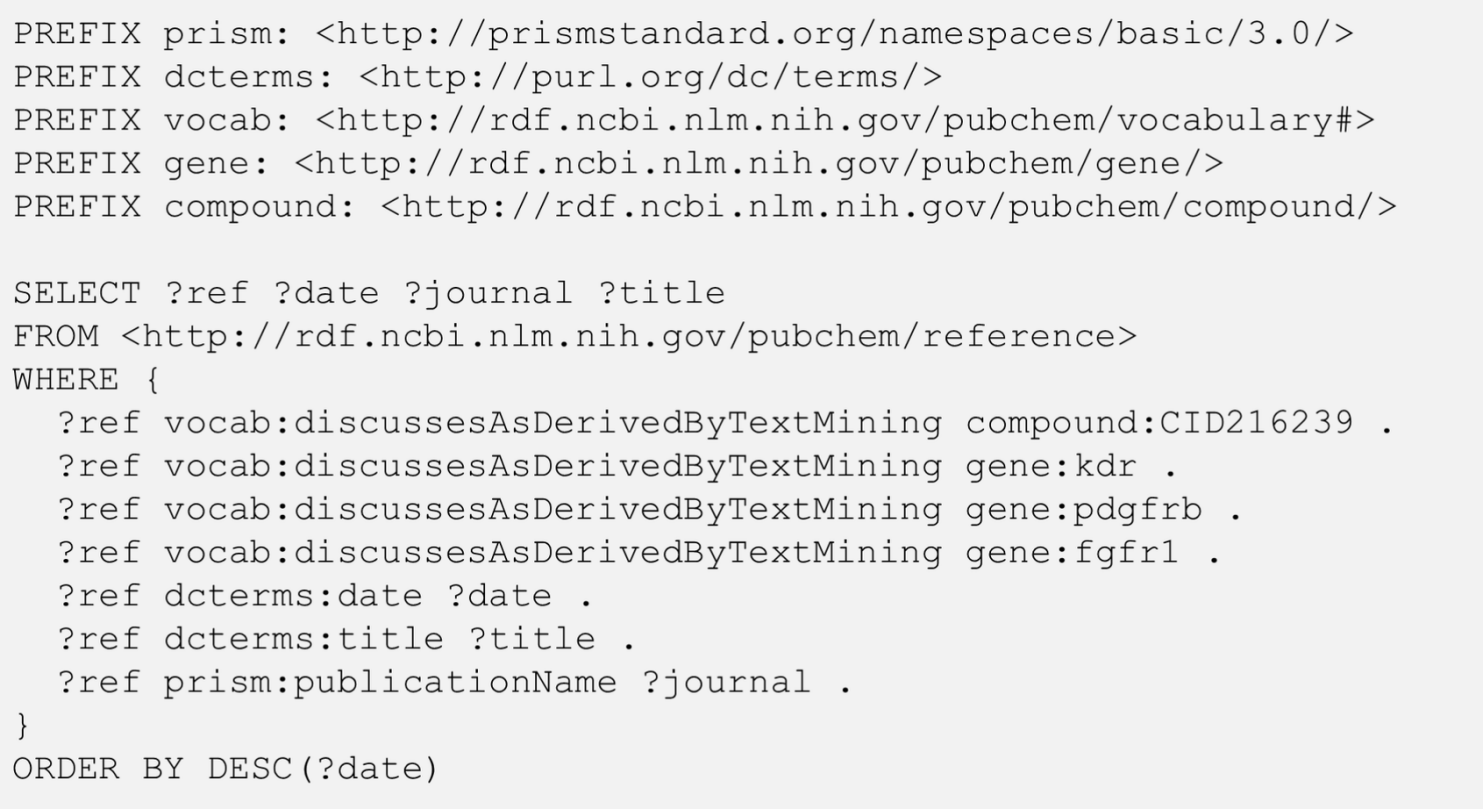
SPARQL query to get the references that simultaneously mention sorafenib (CID 216239) and the three genes: kinase insert domain receptor (KDR), platelet-derived growth factor receptor beta (PDGFRB), and fibroblast growth factor receptor 1 (FGFR1)

The result of the query from Fig. 6 is shown in Table 6. As mentioned previously, the co-occurrence of a drug and all three genes in a single paper is a rare event. Only 23 drugs have been mentioned with all three genes in a single paper. Sorafenib is mentioned with all three genes in four papers, which is the most among the 23 drugs. The four papers co-mentioning sarafenib and the three genes are listed in Table 7. This list was obtained from the query shown in Fig. 7, which is similar to that in Fig. 3.

**Table 6:** Number of references (Num_Refs) that mention chemicals together with all three genes: kinase insert domain receptor (KDR), platelet-derived growth factor receptor beta (PDGFRB), and fibroblast growth factor receptor 1 (FGFR1)

CID	Chemical name	Num_Refs
216239	Sorafenib	4
36314	Paclitaxel	3
9823820	Lenvatinib	3
6137	L-Methionine	2
5329102	Sunitinib	2
25017411	Anlotinib	2
5291	Imatinib	2
426756	Carboplatin	2
11626560	Crizotinib	1
5957	Adenosine-5′-triphosphate	1
876	DL-Methionine	1
5702198	Cisplatin	1
962	Water	1
5329098	Semaxanib	1
6288	Threonine	1
51063134	Methyl cellulose	1
525128	Methyl cellulose	1
5284616	Sirolimus	1
387447	Bortezomib	1
5311	Vorinostat	1
84815	D-Methionine	1
5394	Temozolomide	1
31703	Doxorubicin	1

The data in this Table was returned from the SPARQL query in Fig. 6

**Table 7:** References that mention sorafenib (CID 216239) together with the three genes, kinase insert domain receptor (KDR), platelet-derived growth factor receptor beta (PDGFRB), and fibroblast growth factor receptor 1 (FGFR1), returned from the SPARQL query in Fig. 7

Date	Journal	Title
5/1/2022	Mol. Oncol.	Detection of gene mutations and gene–gene fusions in circulating cell‐free DNA of glioblastoma patients: an avenue for clinically relevant diagnostic analysis
8/12/2016	Expert Opin. Pharmacother.	Efficacy of lenvatinib in treating thyroid cancer
10/1/2012	J. Cell. Mol. Med.	AL3810, a multi-tyrosine kinase inhibitor, exhibits potent anti-angiogenic and anti-tumour activity via targeting VEGFR, FGFR and PDGFR
2/1/2009	Clin. Cancer Res.	Expression of Sorafenib Targets in Melanoma Patients Treated with Carboplatin, Paclitaxel and Sorafenib

### Use case 5: genes co-mentioned with multiple diseases

**Fig. 8:**
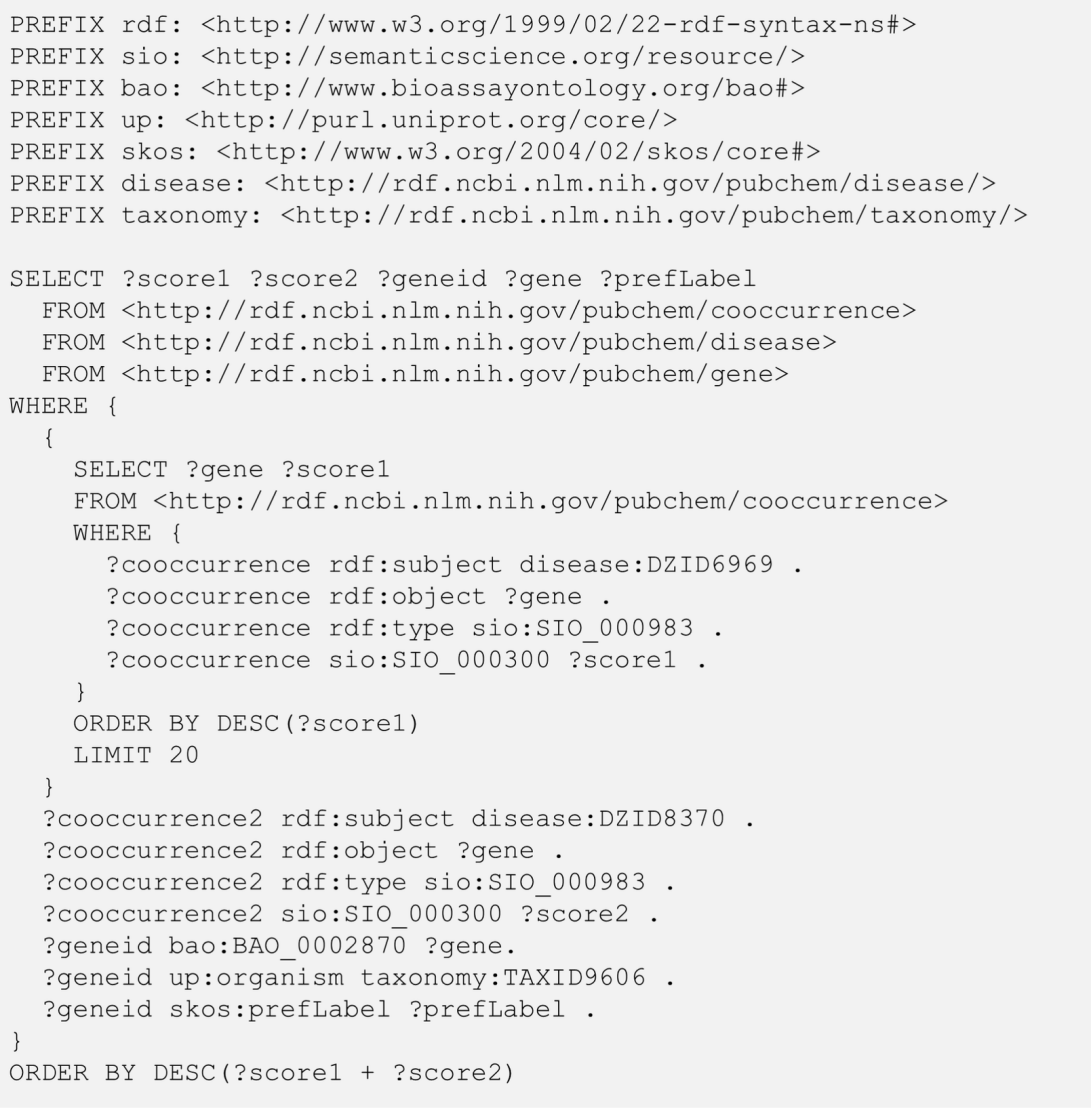
Genes commonly mentioned with two diseases: Rheumatoid Arthritis (DZID 6969) and Systemic Lupus Erythematosus (DZID 8370). The SIO terms SIO_000983 and SIO_000300 mean “gene-disease association” and “has value”, respectively. The RO term, RO_0000087, means “has role”. The BAO term, BAO_0002870, means “has gene symbol”

**Fig. 9:**
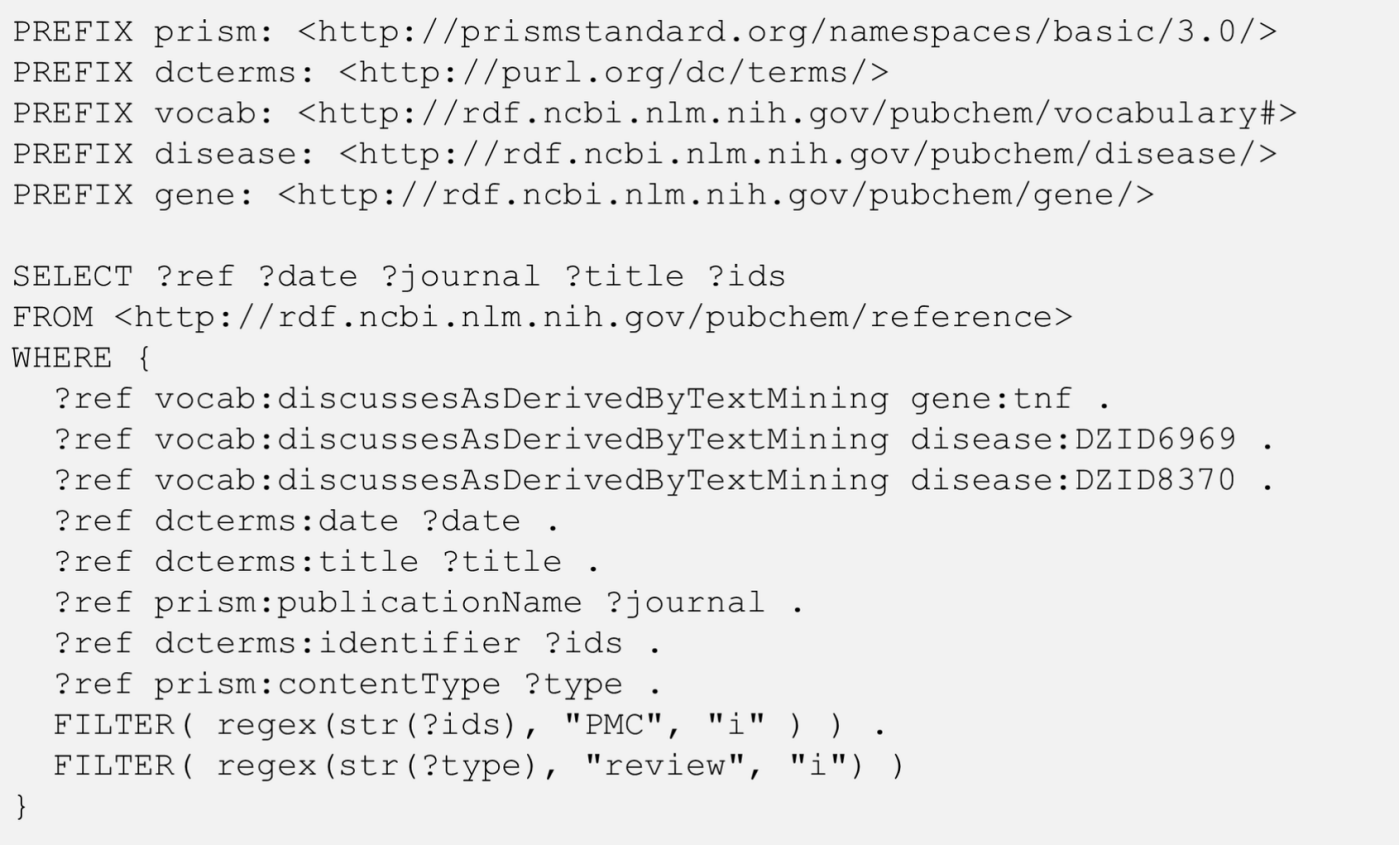
SPARQL query to retrieve review articles that are available within PubMed Central (PMC) that mention the tumor necrosis factor (TNF) gene with rheumatoid arthritis (DZID 6969) and systemic lupus erythematosus (DZID 8370)

The term “comorbidity” refers to multiple disorders or illnesses occurring in the same patient at the same time. In some cases, the comorbidity of two diseases may be attributed to a common gene involved in the pathophysiology of both diseases. The query shown in Fig. 8 identifies the genes co-mentioned with multiple diseases, with rheumatoid arthritis (DZID 6969) and systemic lupus erythematosus (DZID 8370) as an example. This query uses a nested query. The inner query retrieves the top-20 genes co-mentioned with rheumatoid arthritis in terms of co-occurrence score and the outer query refines the resulting genes to only those that are also co-mentioned with systemic lupus arthritis. In the outer query, three lines beginning with “?geneid” get the preferred gene names for the retrieved genes (i.e.,“?gene”). Note that the data underlying the co-occurrence RDF are derived from text mining and that a gene name in the co-occurrence RDF can be associated with multiple genes from different organisms. This redundancy is removed by using the up:organism predicate, which limits the query result only to human genes. The result of the query in Fig. 8 is shown in Table 8. Many of the genes commonly mentioned with rheumatoid arthritis and systemic lupus erythematosus are those involved in immune responses, such as tumor necrosis factor (TNF) and interleukins (ILs). However, the list presented in Table 8 does not provide the context in which the genes are mentioned with the two diseases. This issue may be addressed by finding articles that mention the genes with the two diseases together, as shown in Fig. 9. While the query in Fig. 9 is similar to those in Figs. 3 and 7, it filters the search results to get only those review articles whose full texts are freely available through PubMedCentral (https://www.ncbi.nlm.nih.gov/pmc/). The result from the query is shown in Table 9.

**Table 8:** Genes commonly co-mentioned with two diseases, rheumatoid arthritis (DZID6969) and systemic lupus erythematosus (DZID8370), along with the co-occurrence scores (S_CO_) for the gene-disease pairs

S_CO_		ΣS_CO_	Gene symbol	Gene name
DZID6969	DZID8370			
211406	27828	239234	TNF	Tumor necrosis factor
124538	26360	150898	IL6	Interleukin 6
108809	21325	130134	CRP	C-reactive protein
64147	39853	104000	CD4	CD4 molecule
52343	34474	86817	ACR	Acrosin
61475	17471	78946	IL17A	Interleukin 17A
39171	29949	69120	CD79A	CD79a molecule
44520	24053	68573	IL10	Interleukin 10
54570	5995	60565	PRTN3	Proteinase 3
44651	8894	53545	HLA-DRB1	Major histocompatibility complex, class II, DR beta 1
32928	19650	52578	IL2	Interleukin 2
39802	4272	44074	TNFRSF10A	TNF receptor superfamily member 10a
35127	5600	40727	CXCL8	C-X-C motif chemokine ligand 8
34727	749	35476	TNFSF11	TNF superfamily member 11
33508	1168	34676	PTGS2	Prostaglandin-endoperoxide synthase 2

The genes and co-occurrence scores are returned from the SPARQL query in Fig. 8

**Table 9:** Review articles available within PubMed Central (PMC) that co-mention the TNF gene with two diseases: rheumatoid arthritis (DZID 6969) and systemic lupus erythematosus (DZID 8370)

Date	Journal	Title	PMC ID
3/10/2021	Adv. Rheumatol	Association of homocysteine with ankylosing spondylitis: a systematic review and meta-analysis	PMC7944467
1/1/2010	J. Biomed. Biotechnol	Anti-TNF-α therapies in systemic lupus erythematosus	PMC2896679
1/1/2010	Curr. Dir. Autoimmun	Type I Interferon: A New Player in TNF Signaling	PMC2827816
10/15/2008	Arthritis Res. Ther	Translating co-stimulation blockade into clinical practice	PMC2582811

The articles and associated data are returned from the SPARQL query in Fig. 9

### Interoperability of co-occurrence RDF data

Chemicals, genes, and diseases in PubChem are annotated with the identifiers for relevant records in information resources external to PubChem. These annotations are also encoded in RDF using appropriate predicates. For example, chemicals are annotated with the identifiers used in ChEBI [32], National Drug File Reference Terminology (NDFRT) [33], SNOMED-CT (Systematized Nomenclature of Medicine-Clinical Terms) [34], and NCI Thesaurus (NCIt) [35] through predicates vocab:is_active_ingredient_of, rdf:type, and rdfs:seeAlso, as shown in the following examples:

- https://pubchem.ncbi.nlm.nih.gov/rest/rdf/query?graph=compound&subject=compound:CID60823&predicate=rdf:type
- https://pubchem.ncbi.nlm.nih.gov/rest/rdf/query?graph=compound&subject=compound:CID60823&predicate=rdfs:seeAlso
- https://pubchem.ncbi.nlm.nih.gov/rest/rdf/query?graph=compound&subject=compound:CID60823&predicate=vocab:is_active_ingredient_of

Note that the above examples also demonstrate how to access PubChemRDF data through a Representational State Transfer (REST)-ful interface [36] (see the PubChemRDF REST-ful Interface page for more details about programmatic access to PubChemRDF data through the REST-ful interface).

Diseases in PubChemRDF are also linked to other information resources, including PharmGKB [37], MedGen [38], IUPHAR/BPS Guide to Pharmacology [39], Unified Medical Language System (UMLS) [40], Kyoto Encyclopedia of Genes and Genomes (KEGG) Disease [41], NCI Thesaurus [35], MeSH [23], MONDO Disease Ontology [42], Nanbyo Disease Ontology (NANDO), Orphanet [43], and Human Disease Ontology [44]. The “closeMatch” and “relatedMatch” predicates are used to link disease records between the resources, as shown in the following examples (for Parkinson’s disease (DZID8805)).

- https://pubchem.ncbi.nlm.nih.gov/rest/rdf/query?graph=disease&subject=disease:DZID8805&predicate=skos:closeMatch
- https://pubchem.ncbi.nlm.nih.gov/rest/rdf/query?graph=disease&subject=disease:DZID8805&predicate=skos:relatedMatch

As mentioned in the “Uniformed Resource Identifiers for Named Entities” section, a gene entity in the co-occurrence RDF is specified with its (case-insensitive) gene symbol, which can be associated with multiple genes from different species. In contrast, many gene information resources treat these orthologous genes as distinct records to provide species-specific data. Therefore, linking a gene in the co-occurrence RDF with corresponding records in other resources requires an intermediate step in which the gene symbol is resolved to a species-specific record in the NCBI Gene database [45]. Non-species-specific gene symbols have incoming links from species-specific NCBI Gene identifiers, as shown in this example (for the breast cancer 1 (brca1) gene):

- https://pubchem.ncbi.nlm.nih.gov/rest/rdf/query?graph=gene&predicate=bao:BAO_0002870&object=gene:brca1

where the “bao:BAO_0002870” predicate means “has gene symbol”. The above request returns the list of gene records (represented by NCBI Gene IDs) whose gene symbol is “brca1” (case-insensitive), including Gene IDs 672 (for human), 827854 (for thale cress), 497672 (Norway rat), 733513 (tropical clawed frog), 12189 (house mouse), and 353120 (domestic cattle). These Gene IDs can be used to get related records in other databases, as shown in this example for Gene ID 672:

- https://pubchem.ncbi.nlm.nih.gov/rest/rdf/query?graph=gene&subject=gene:GID672&predicate=rdfs:seeAlso

Through the rdfs:seeAlso predicate, the gene records are linked to related records in other sources, including Open Targets [46], NCBI Gene [45], Ensemble [47], UniProt [48], MedlinePlus [49], the Gene Curation Coalition (GenCC) [50], Comparative Toxicogenomics Database (CTD) [51], Pharos [52], MeSH [23], NCI thesaurus (NCIt) [35], Bgee [53], Alliance of Genome resources [54], KEGG Gene [41], PharmGKB [37], Online Mendelian Inheritance in Man (OMIM) [55], Expasy [56], Human Genome Organization (HUGO) Gene Nomenclature Committee (HGNC) [57], VEuPathDB [58], Mouse Genome Informatics (MGI) [59], Rat Genome Database (RGD) [60], and XenBase [61]. The gene records are also linked to Gene Ontology terms through the “participates in” (RO_0000056), “has function” (RO_0000085), and “located in” (RO_0001025) predicates from the Open Biological and Biomedical Ontologies (OBO) Relations Ontology (RO) [62]:

- https://pubchem.ncbi.nlm.nih.gov/rest/rdf/query?graph=gene&subject=gene:GID672&predicate=obo:RO_0000056
- https://pubchem.ncbi.nlm.nih.gov/rest/rdf/query?graph=gene&subject=gene:GID672&predicate=obo:RO_0000085
- https://pubchem.ncbi.nlm.nih.gov/rest/rdf/query?graph=gene&subject=gene:GID672&predicate=obo:RO_0001025

### Limitations

The limitations of co-occurrence data are detailed in our previous paper [4]. Because named entities were extracted from PubMed records using the LeadMine text-mining software [8], the quality of the co-occurrence data is directly tied to the accuracy of LeadMine. LeadMine uses expertly curated grammars and dictionaries, along with dictionaries automatically derived from public resources. It showed 89.9% precision and 85.4% recall, resulting in 87.6% F1-score, on the CHEMDNER test set [8].

The co-occurrence data used in this study does not capture the context in which two entities are co-mentioned. For example, while both sorafenib (CID216339) and 2,3,7,8-tetrachlorodibenzo-p-dioxin (TCDD) (CID15625) are frequently mentioned with hepatocellular carcinoma (DZID7998), sorafenib is an anti-cancer drug but TCDD is a carcinogen. Therefore, it is highly recommended to retrieve the papers mentioning entities of interest (as shown in Use Case 2) and check the context of the entity relationships.

As mentioned in the “RDF Data Model” section, the co-occurrence data does not differentiate between proteins and their encoding genes, but treats them all as genes, reflecting their frequent interchangeable use in the literature. As a result, the co-occurrence RDF data model does not encode relationships of proteins with chemicals, diseases, and other proteins. However, the relationships between proteins and their encoding genes are already encoded in PubChemRDF protein subdomain, using the UniProt “encodedBy” predicate as in this example for Gene ID 672:

- https://pubchem.ncbi.nlm.nih.gov/rest/rdf/query?graph=protein&predicate=up:encodedBy&object=gene:GID672

This allows users to extend gene-based co-occurrence relationships to protein-based ones by converting gene symbols to species-specific Gene IDs and subsequently to their encoded proteins. However, this extension introduces uncertainty because mentions of gene names in the literature often lack organism information. Additionally, the fact that a single gene can encode multiple proteins, resulting in one-to-many relationships, adds further ambiguity.

## Conclusions

In this paper, we described an RDF data model that expresses co-occurrence associations between chemicals, genes, and diseases derived from biomedical literature. This data model allows users to quickly identify chemicals, genes/proteins, and diseases mentioned together with a given named entity (Use Case 1). In addition, the model can be used to get references that mention two entities together, helping one to understand the context of the co-occurrence association between the entities (Use Case 2). It can also be used to find an implicit link between entities that are not mentioned together, through the common entities associated with them (Use Case 3). The co-occurrence RDF can be used to perform more complicated tasks. For example, Use Case 4 demonstrates how to identify chemicals co-mentioned with multiple genes, which may be applicable for multi-target ligand discovery. Use Case 5 explains how to find genes co-mentioned with multiple diseases to identify genes that are potentially responsible for the disease comorbidity.

The underlying data used in the co-occurrence RDF was derived from text mining of millions of references available in PubMed using the approaches described in our previous study [4]. While this data is also used to generate the PubChem literature knowledge panel, the co-occurrence RDF enables additional tasks. For example, with the co-occurrence RDF, the user can work with a large set of relevant co-occurrence neighbors (up to 1000) for a given entity and automate this data retrieval task using a computer program or script. The co-occurrence RDF model is an enhancement to the PubChemRDF ecosystem that can facilitate exploring biomedical knowledge and seeking new discoveries in a semantic way. More importantly, it is naturally connecting to other linked data resources in various scientific communities to greatly enhance the usability and accessibility of biomedical data. The co-occurrence RDF data as well as PubChem data are routinely updated through an automated pipeline. The co-occurrence RDF data used in this study is freely available at Zenodo (10.5281/zenodo.10126725) [26].

## Supplementary Information

Additional file 1. A tab-separated-values (TSV) file containing chemicals mentioned in this paper.

## Data Availability

The latest version of the co-occurrence RDF data and other PubChemRDF data can be accessed via the PubChem FTP site (https://ftp.ncbi.nlm.nih.gov/pubchem/RDF/). The up-to-date RDF schema in various formats is available on the PubChemRDF Schema page (https://pubchem.ncbi.nlm.nih.gov/docs/rdf-schema). A set of SPARQL query examples can be found on the PubChemRDF use case pages (https://pubchem.ncbi.nlm.nih.gov/docs/rdf-use-cases). For archival purposes, the co-occurrence RDF data generated in this study, along with the SPARQL query examples, the RDF schema in SHACL [24] and ShEx [25], and the validation scripts are freely available at Zenodo (10.5281/zenodo.10126725) [26].
